# Not Your Typical Bisphosphonate Intolerance: A Case of Unusual Bone Pain With Low Alkaline Phosphatase

**DOI:** 10.7759/cureus.23163

**Published:** 2022-03-14

**Authors:** Brant W Bickford, Sonia Bennett, Ronald J Markert

**Affiliations:** 1 Wright State University Internal Medicine Residency Program, Wright-Patterson Air Force Base, Dayton, USA; 2 Department of Endocrinology, Wright State University, Dayton, USA; 3 Department of Medicine, Wright State University, Dayton, USA

**Keywords:** tissue-nonspecific alkaline phosphatase, bone pain, decreased alkaline phosphatase, bisphosphonate use, hypophosphatasia

## Abstract

Bisphosphonates, first-line medications for osteoporosis, are often not tolerated or discontinued for multiple reasons. Hypophosphatasia (HPP) is a genetic deficiency with the enzyme activity of tissue-nonspecific alkaline phosphatase (TNSALP). The symptoms of adult HPP are often non-specific, and the diagnosis may be delayed for years. Low serum alkaline phosphatase, a hallmark feature of HPP, is often overlooked. Genetic testing is recommended to confirm diagnosis, and treatment with asfotase alfa, a recombinant alkaline phosphatase, is available for patients with HPP. We report a case of HPP in a 71-year-old female with recurrent skeletal pain and bisphosphonate intolerance who ultimately was diagnosed with HPP.

## Introduction

Hypophosphatasia (HPP) is a rare genetic deficiency with the enzyme activity of tissue-nonspecific alkaline phosphatase (TNSALP). Low TNSALP activity leads to extracellular accumulation of substrates that lead to bone mineralization defects and systemic complications. While not firmly established, the prevalence of hypophosphatasia in the United States is estimated to be approximately 1 in 300,000 [1]. However, given the variability of symptoms and the large number of undiagnosed cases, the prevalence of adult HPP is difficult to estimate [2]. Currently, nearly 500 ALPL mutations in the gene encoding TNSALP have been reported in the ALPL gene database with variable levels of expression [3]. Six different clinical variants have been reported, defined by the age of onset and characterized by different degrees of severity.

Adult HPP patients may present with symptoms of early loss of primary and secondary teeth, stress fractures of the metatarsals, atypical femoral fractures, joint pain, skeletal deformity, and functional impairment in gait and mobility. Unfortunately, the diagnosis of HPP is often delayed in adults by as much as 10 years while the manifestations occur at a median age of 37.6 years [4]. Low serum alkaline phosphatase is a hallmark laboratory feature that is often overlooked during the presentation.

Dual-energy X-ray absorptiometry (DEXA) scans in HPP patients have the typical features of osteoporosis, and patients may be started on bisphosphonates (BP) which actually leads to further extracellular accumulation of substrates and worsen symptoms. Moreover, providers may discontinue BP based on common side effects of therapy such as gastrointestinal (GI) problems, atypical femur fractures, musculoskeletal pain, and may not investigate these worsening symptoms further.

This case was previously presented as an endocrine breakout podium presentation at the Air Force American College of Physicians Meeting in September 2021.

## Case presentation

A 71-year-old female with a past medical history of osteoporosis, vitamin B6 elevation (discovered at a work health screening event), and hypertension presented to an outpatient endocrinology clinic with chief complaints of generalized weakness and “bone pain” of six-month duration. She noted that many years of bone pain worsened with BP therapy without explanation. Her most recent flare lasted six months and correlated with her denosumab injection. She denied history of fractures, kidney stones, injuries, muscle aches, dental problems, or early tooth loss. Family history was only significant for mother with traumatic hip fracture at a young age. The physical examination was benign, and vitals were within normal limits. Her muscle weakness was primarily axial in nature, but all strength testing was 5/5 without asymmetric deficit. No gum/dental abnormalities were identified.

The laboratory findings were significant for serum alkaline phosphatase 34 U/L (38-126) with a repeat being 22 U/L. Other components of her comprehensive medical panel were within normal limits. Additional labs specific to HPP are below in Table 1. Most recent DEXA scan was in 2019 with T-scores of -2.3 for L1-L4, -2.2 for left femoral neck, and -2.4 for right femoral neck, with an elevated FRAX risk [5].

**Table 1:** Lab Investigations

Test	Result	Reference
Alkaline Phosphatase	34 U/L	38-126 U/L
Pyridoxine (Vitamin B6)	308.6 nmol/L	20-125 nmol/L
Urine Phosphoethanolamine	201 micromol/g	40-155 micromol/g
Vitamin D	47 ng/mL	30-100 ng/mL
Calcium	9.8 mg/dL	8.5-10.5 mg/dL
Albumin	7.2 g/dL	3.4-5.4 g/dL
Parathyroid Hormone	35.3 pg/mL	15-65 pg/mL

We recommended genetic testing to the patient, but she declined. Subsequently, she was instructed to continue vitamin D and calcium supplementation and to forgo any additional bisphosphonate or denosumab injections as these would likely worsen her symptoms. Follow-up DEXA in 2021 revealed improvement in the lumbar spine with T score of -1.8, and the right femoral neck also improved to T score of -1.6. The patient’s bone pain remained bothersome at follow-up and she is in the process of starting asfotase alfa therapy.

## Discussion

The adult presentation of HPP is variable with non-specific symptoms including bone and muscle pain, joint issues, fractures, and dental issues. These symptoms are often misidentified as osteoporosis or osteoarthritis. Unfortunately, our patient suffered for an extended time despite having a low serum alkaline phosphatase for years. She had seen multiple specialists for her chronic pain with the last being a rheumatologist prior to her referral to endocrinology for low serum alkaline phosphatase. Low alkaline phosphatase is a rare and often unrecognized reason for provider concern [6].

The diagnosis of HPP can be made with an appropriate history, physical exam, laboratory studies, and radiographic findings. Persistently low alkaline phosphatase is a hallmark of HPP. Additional labs include serum pyridoxal-5’-phosphate (vitamin B6) and urine phosphoethanolamine, both of which were abnormally elevated with our patient [7]. Physicians must be aware that the normal range for alkaline phosphatase varies based on age and gender. Alkaline phosphatase levels can be decreased in several conditions, with recent literature supporting reflex testing of low serum alkaline phosphatase with pyridoxal-5-phosphate (PLP) given a higher specificity for HPP. Additionally, both decreased alkaline phosphatase and elevated PLP can correlate with disease activity [8].

A limitation of our case report is that our patient decided not to pursue genetic testing. While not needed for the diagnosis, genetic testing would have further clarified her type of mutation. Most mutations are missense with autosomal dominant and autosomal recessive inheritance patterns. Genetic testing has some utility for the assessment of risk for future generations [9]. Treatment is available for HPP patients with asfotase alfa, a recombinant alkaline phosphatase. Bisphosphonates and denosumab should be avoided since they can further lower alkaline phosphatase and alter skeletal mineralization [10]. Traditional bone health practices, including adequate vitamin D levels and weight-bearing activity, are recommended.

Asfotase alfa is a human recombinant tissue-nonspecific alkaline phosphatase fusion protein with enzymatic activity that promotes bone mineralization in patients with HPP. Initially approved for the treatment of perinatal and infantile forms of HPP in 2015, treatment of adult onset HPP with asfotase alfa is increasing with case reports demonstrating improved pedometer step counts and bone mineralization [11]. Given recent FDA approval and utilization of treatment in adult onset HPP, little long-term data currently exists. However, there are reports of improvement of gross motor function, muscle strength, improvement of patient-reported functional disability over five years of treatment [12].

## Conclusions

Hypophosphatasia is a rare genetic condition with skeletal defects that may present with non-specific bone complaints, which may include bisphosphonate intolerance. Adult presentation of HPP is often non-specific and can take years to diagnose and our case was unique given her chief complaint of "bone pain" and age of diagnosis. Hallmark features include low serum alkaline phosphatase and elevated vitamin B6 level, and elevated urine phosphoethanolamine. Genetic testing should be performed to confirm the diagnosis and risk assessment for future generations. Recombinant alkaline phosphatase therapy with asfotase alfa treatment is available for patients and bisphosphonates should be avoided.
